# SIRT1-dependent epigenetic regulation of H3 and H4 histone acetylation in human breast cancer

**DOI:** 10.18632/oncotarget.25771

**Published:** 2018-07-17

**Authors:** Khaldoun Rifaï, Gaëlle Judes, Mouhamed Idrissou, Marine Daures, Yves-Jean Bignon, Frédérique Penault-Llorca, Dominique Bernard-Gallon

**Affiliations:** ^1^ Department of Oncogenetics, Centre Jean Perrin, CBRV, Clermont-Ferrand 63001, France; ^2^ INSERM, UMR 1240, IMoST Imagerie Moléculaire et Stratégies Théranostiques, Clermont-Ferrand 63005, France; ^3^ Department of Biopathology, Centre Jean Perrin, Clermont-Ferrand 63011, France

**Keywords:** breast cancer, epigenetics, SIRT1, histone marks, epigenetic modulation

## Abstract

Breast cancer is the most frequently diagnosed malignancy in women worldwide. It is well established that the complexity of carcinogenesis involves profound epigenetic deregulations that contribute to the tumorigenesis process. Deregulated H3 and H4 acetylated histone marks are amongst those alterations. Sirtuin-1 (SIRT1) is a class-III histone deacetylase deeply involved in apoptosis, genomic stability, gene expression regulation and breast tumorigenesis. However, the underlying molecular mechanism by which SIRT1 regulates H3 and H4 acetylated marks, and consequently cancer-related gene expression in breast cancer, remains uncharacterized. In this study, we elucidated SIRT1 epigenetic role and analyzed the link between the latter and histones H3 and H4 epigenetic marks in all 5 molecular subtypes of breast cancer. Using a cohort of 135 human breast tumors and their matched normal tissues, as well as 5 human-derived cell lines, we identified H3k4ac as a new prime target of SIRT1 in breast cancer. We also uncovered an inverse correlation between SIRT1 and the 3 epigenetic marks H3k4ac, H3k9ac and H4k16ac expression patterns. We showed that SIRT1 modulates the acetylation patterns of histones H3 and H4 in breast cancer. Moreover, SIRT1 regulates its H3 acetylated targets in a subtype-specific manner. Furthermore, SIRT1 siRNA-mediated knockdown increases histone acetylation levels at 6 breast cancer-related gene promoters: *AR*, *BRCA1*, *ERS1*, *ERS2*, *EZH2* and *EP300*. In summary, this report characterizes for the first time the epigenetic behavior of SIRT1 in human breast carcinoma. These novel findings point to a potential use of SIRT1 as an epigenetic therapeutic target in breast cancer.

## INTRODUCTION

Breast cancer remains the leading cause of cancer death among females in less developed countries, and second leading cause of cancer death in more developed countries after lung cancer [1]. The occurrence of breast cancer is a complex, multifactorial process that is regulated by a number of different genes at different tumor formation stages [2]. Breast cancer is also characterized by its molecular and clinical heterogeneity with variations in gene expression profiles among women [3]. The St. Gallen molecular classification divides breast tumors into 5 distinct subtypes in ascending order of tumor aggressiveness [4]. Luminal A, luminal B (HER2-) and luminal B (HER2+), these 3 subtypes are included in the Hormone Receptor-positive Breast Cancer (HRBC). HER2-enriched or HER2 Breast Cancer (H2BC). And finally triple-negative breast cancer (TNBC), also known as basal-like, which is characterized as very aggressive compared to the other molecular subtypes [5]. 85 to 90% of breast tumors are called sporadic or non-hereditary tumors that can spawn due to many environmental risk factors. Sporadic breast tumors are especially characterized by the presence of underlying abnormalities in their epigenome [6].

The complexity of carcinogenesis cannot be represented by genetic mutations alone, but also involves profound epigenetic alterations. The epigenetic regulation of the genome includes among others, histone post-translational modifications (PTMs) [7]. Deregulated histone PTMs or histone marks are considered as biomarkers of cancer prognosis and were shown to predict patient outcome in various human carcinomas [8, 9]. In breast cancer, analysis of human breast tumors revealed a highly significant correlation between global histone marks patterns and tumor molecular phenotypes, prognostic factors, and clinical outcome [10, 11]. Lysine acetylation at the N-terminus tails of histones H3 and H4 is classically associated with increased gene expression. The epigenetic marks (epi-marks) H3 lysine 4 (H3K4ac), lysine 9 (H3K9ac) and H4 lysine 16 (H4K16ac) are well-characterized acetylated marks that are particularly enriched at transcriptionally active gene promoters [12] [13]. H3K9ac and H4K16ac have well-defined roles in regulating chromatin structure. Their deacetylation causes the formation of higher-order chromatin compaction and subsequently transcription repression, as neatly described by Vaquero *et al.* [14, 15]. Global loss of H4K16ac has been shown to be a hallmark of human cancer and associated with early tumor formation stages [16]. Also, H3K9ac has been shown to be underexpressed in breast cancer, as well as other cancers, and its decrease was correlated with tumor progression and poor clinical outcome [10]. On the other hand, few reports studied the role of H3K4ac in cancer. The function of H3K4ac was often related to that of H3K4me3, since both acetylation and methylation of lysine (K4) residue are associated with active transcription [17]. In addition, the epigenetic acyl-lysine ‘eraser’ of H3K4ac histone marker has not been yet identified in humans. In a recent study, Messier *et al.* explored the dynamics of H3K4ac in 2 breast cancer cell lines. They demonstrated the latter as an indicator of deregulated cancer-related pathways. They also uncovered a role of H3K4ac in predicting epigenetic changes associated with early stages of transformation [18].

Histone deacetylases (HDACs) are major actors of epigenetic regulation. Dysfunctional HDACs have been found to be closely related to the tumorigenesis process and cancer metastasis [19]. Due to their deacetylase activity of a broad spectrum of substrates, Sirtuins are considered to be master regulators of several basic cellular mechanisms [20]. Silent mating type information regulation 2 homolog 1 (SIRT1) is a NAD+-dependent class III HDAC. The founding member of the Sirtuins family is tightly implicated in the regulation of numerous key cellular processes including apoptosis and cell survival, DNA damage repair, chromatin remodeling, gene expression regulation, and cancer development and metastasis [20, 21]. It has been shown that SIRT1 regulates genome stability in part through deacetylation of N-terminus tails of acetylated histones: H1K26ac, H3K56ac, H2A variant H2A.Z, in addition to H3K9ac and H4K16ac [14, 15, 22]. SIRT1 also regulates the catalytic activity of a plethora of downstream non-histone targets. For example, SIRT1 can deacetylate and downregulate the activity of tumor suppressor enzymes such as p53 [23], p73 [24], E2F1 [25], and Forkhead box proteins FOXO transcription factors [26], but also oncogenes such as NF-kappaB [27], STAT3 [28], Survivin [29] and β-Catenin [30]. On the contrary, SIRT1 can upregulate the activity of other oncogenes like c-Myc [31] and HIF-1α [32]. As a result, the critical role of multifaceted SIRT1 in human carcinogenesis remains very controversial due to its contradictory functional roles [33, 34]. In breast cancer, both tumor-suppressive and tumor-promoting functions of SIRT1 have been reported and the controversy regarding SIRT1 role in the disease continues still.

SIRT1 implication in the occurrence and progression of breast cancer pathogenesis have been identified and extensively investigated over recent years. However, SIRT1-dependent epigenetic regulation of H3 and H4 acetylated histone marks, and consequently cancer-related gene expression in human breast cancer, has not been investigated yet. In this study, we examined for the first time the epigenetic mechanisms by which SIRT1 regulates the acetylation patterns of histones H3 and H4 epigenetic marks in sporadic breast cancer, we also investigated the link between SIRT1 and the 3 epi-marks H3K4ac, H3K9ac and H4K16ac in all 5 intrinsic subtypes of the disease. The present report adds a layer of clarity on the ongoing controversy of SIRT1 behavior in human breast carcinoma.

## RESULTS

### Description of the study cohort characteristics

The breast cancer molecular subtypes studied here spanned luminal A (*n* = 36, 26.7%), luminal B (HER2-) (*n* = 34, 25.2%), luminal B (HER2+) (*n* = 25, 18.5%), HER2- enriched (*n* = 15, 11.1%) and triple-negative breast cancer (TNBC) (*n* = 25, 18.5%) (Table 1). All patients were females aged 40 to 84 years (mean 64.6 ± SD 5.3). All tumors were graded according to the modified Scarff- Bloom-Richardson grading system (SBR) as grade 1 (*n* = 17), grade 2 (*n* = 69) and grade 3 (*n* = 49). Tumor size ranged from 0.4 to 7.8 cm (3.1 ± 0.7). Samples were ER-, PR- and HER2-positive in *n* = 95 (70.3%), *n* = 58 (42.9%) and *n* = 40 (29.6%) patients, respectively. We found an insignificant correlation between all intrinsic subtypes and age of patients (*p* = 0.643) and tumor size (*p* = 0.079). Luminal A and B (HER2-) subtypes presented a significant correlation with low SBR grade tumors, whereas luminal B (HER2+), HER2- enriched and triple-negative subtypes exhibited high SBR grade tumors (*p* < 0.001). As for the hormonal receptors status, a clear distinction between the 5 molecular subtypes can be noted as per the molecular classification of St. Gallen. The clinico-pathological variables of the 135 breast cancer patients are presented in (Table 1).

**Table 1:** Clinico-pathological characteristics of the breast cancer patients included in this study

	Total	Luminal A	Luminal B (HER2-)	Luminal B (HER2+)	HER2-enriched	Triple-negative	*P* value^†^
Patients, *n* (%)	*N* = 135 (100%)	*n* = 36 (26.7%)	*n* = 34 (25.2%)	*n* = 25 (18.5%)	*n* = 15 (11.1%)	*n* = 25 (18.5%)	
Age at diagnosis							0.643
45–65	66 (48.8)	21 (58.3)	15 (44.1)	12 (48)	8 (53.3)	10 (40)	
> 65	69 (51.2)	15 (41.6)	19 (55.8)	13 (52)	7 (46.6)	15 (60)	
SBR grade							**0.0001**
I	17 (12.5)	13 (36.1)	4 (11.7)	0	0	0	
II	69 (51.1)	21 (58.3)	24 (70.5)	11 (44)	6 (40)	7 (28)	
III	49 (36.4)	2 (5.5)	6 (17.6)	14 (56)	9 (60)	18 (72)	
Size of tumor (cm)							0.079
< 1.5	31 (22.9)	11 (30.5)	9 (26.4)	5 (20)	2 (13.3)	4 (16)	
1.5–2.5	61 (45.1)	19 (52.7)	18 (52.9)	11 (44)	5 (33.3)	8 (32)	
> 2.5	43 (31.8)	6 (16.6)	7 (20.5)	9 (36)	8 (53.3)	13 (52)	
ER status (%)							**0.0001**
Positive	95 (70.3)	36 (100)	34 (100)	25 (100)	0	0	
Negative	40 (29.6)	0	0	0	15 (100)	25 (100)	
PR status (%)							**0.0001**
0%–50%	20 (14.8)	7 (19.4)	8 (23.5)	5 (20)	0	0	
51%–100%	38 (28.1)	29 (80.5)	7 (20.5)	2 (8)	0	0	
Negative	77 (57)	0	19 (55.8)	18 (78)	15 (100)	25 (100)	
HER2 status (%)							**0.0001**
Positive	40 (29.6)	0	0	25 (100)	15 (100)	0	
Negative	95 (70.3)	36 (100)	34 (100)	0	0	25 (100)	
Ki67 status (%)							**0.0001**
≤ 20%	53 (39.2)	29 (80.5)	8 (23.5)	7 (28)	4 (26.6)	5 (20)	
> 20%	82 (60.7)	7 (19.4)	26 (76.4)	18 (72)	11 (73.3)	20 (80)	

Abbreviations: ER: Estrogen Receptor, PR: Progesterone Receptor, HER2: Human Epidermal growth factor Receptor 2, Ki-67: cellular marker for proliferation.

^†^Pearson’s Chi-square test.

### Inverse correlation between SIRT1 and H3k4ac, H3k9ac and H4k16ac global expression patterns in breast tumors versus matched normal tissues

**Figure 1:**
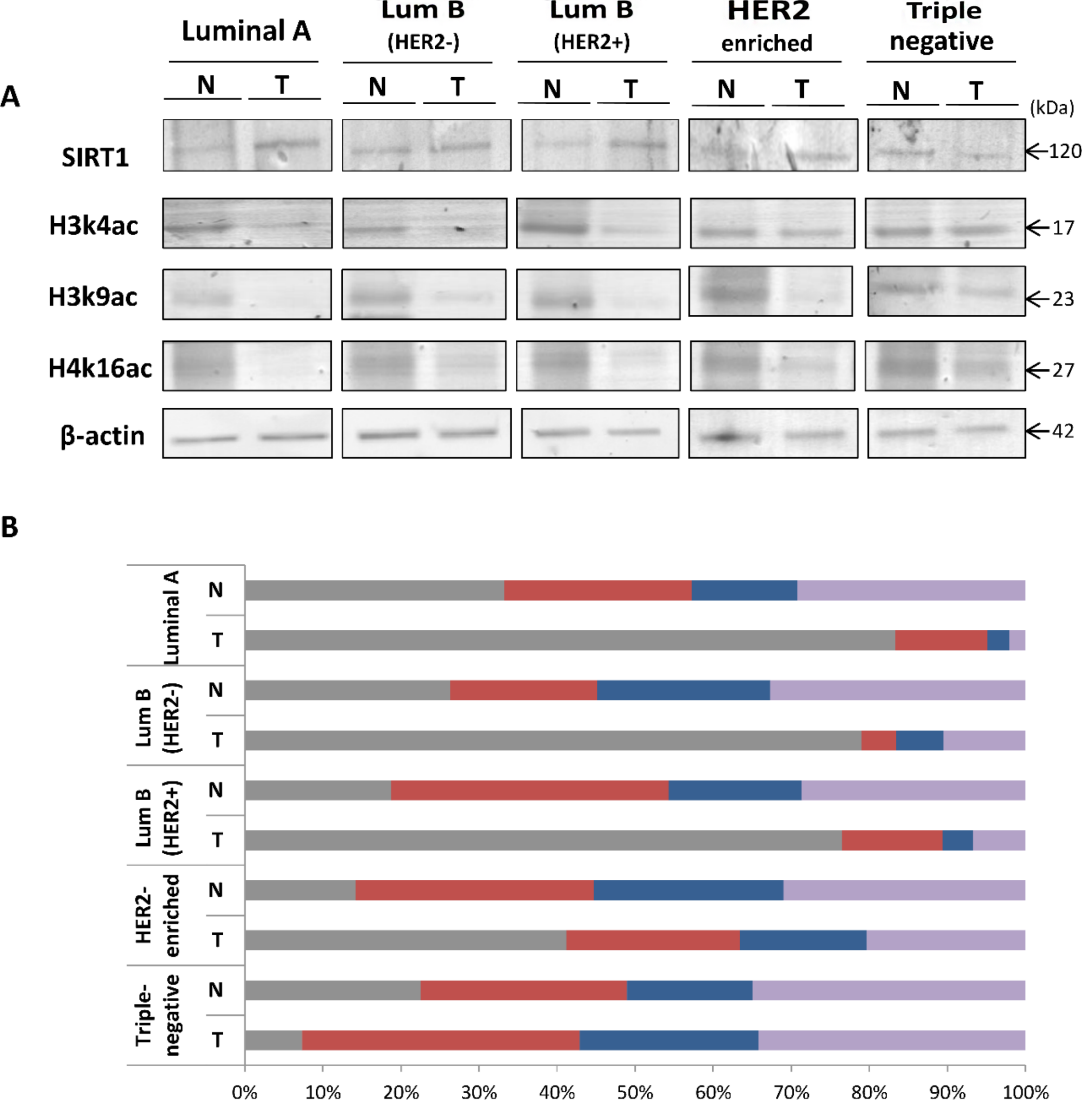
Differential expression patterns of SIRT1, H3k4ac, H3k9ac and H4k16ac in the 5 molecular breast tumor subtypes compared to matched normal tissues (**A**) Equal amounts of proteins were immunoblotted with anti-SIRT1 Ab (120 kDa), anti-H3k4ac Ab (17 kDa), anti-H3k9ac Ab (23 kDa) and anti-H4k16ac Ab (27 kDa). β-actin (42 kDa) served as an internal loading control. (**B**) Relative expression levels were evaluated using Quantity One software and normalized against the internal control β-actin. Each bar represents the percentage contribution of each of the 4 proteins compared to the total set as (100%). All experiments were performed in triplicate fashion. N: Normal, T: Tumor.

In order to investigate the epigenetic role of the histone deacetylase SIRT1 in sporadic breast cancer, we began our studies in *ex-vivo* by assessing the relative expression levels of SIRT1 and the 3 epigenetic marks H3k4ac, H3k9ac and H4k16ac in all 5 molecular subtypes of breast tumors and their matched normal tissue samples using immunoblot analysis (Figure 1A). The blots showed a significant upregulation of SIRT1 expression levels in luminal and HER2-enriched subtypes and significant downregulation in TNBC subtype, in comparison with their matched normal tissues (Figure 1B). The differential expression pattern of SIRT1 across the 5 molecular subtypes was characterized in our earlier study [35]. In contrast, the expression levels of H3k4ac, H3k9ac, and H4k16ac were significantly reduced in luminal and HER2-enriched subtypes and relatively upregulated in TNBC subtype, all compared to their matched normal tissues (Figure 1B). This inverse correlation provides a causal link between the expression patterns of SIRT1 and the 3 epi-marks in human breast cancer.

### SIRT1 simultaneously colocalizes and physically interacts with histone H3 acetylated marks in human breast cancer

**Figure 2:**
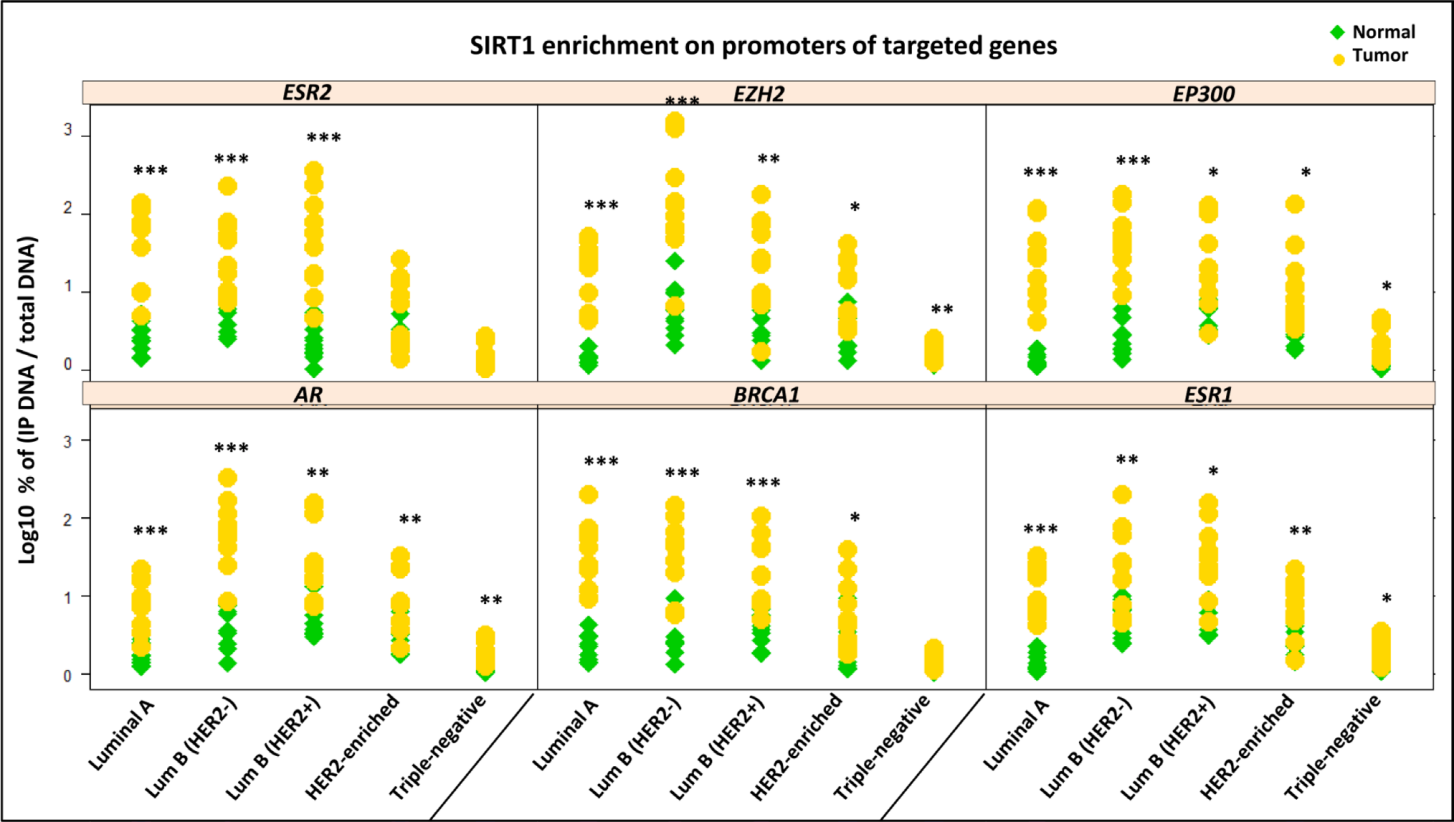
SIRT1 enrichment on promoters of 6 breast cancer-related genes in the 5 molecular breast tumor subtypes versus matched normal tissues Column scatter plot showing the results of ChIP assays using anti-SIRT1 Ab on 50 breast tumors and their 50 matched normal tissues: *n* = 10 tumors for each of the 5 molecular subtypes. The efficiency of ChIP was calculated by real time quantitative PCR using the primers and probes of 6 targeted genes: *AR, BRCA1, ERS1, ERS2, EZH2* and *EP300.* The y-axis represents the log expression percent of (IP DNA/Total DNA) on target genes promoters. Statistically significant difference of SIRT1 enrichment in tumors versus normal tissues was analyzed by Student’s *t*-test. *P* values were two-tailed, ^*^P < 0.05, ^**^P <0.01 and ^***^P < 0.001 were considered statistically significant.

**Figure 3:**
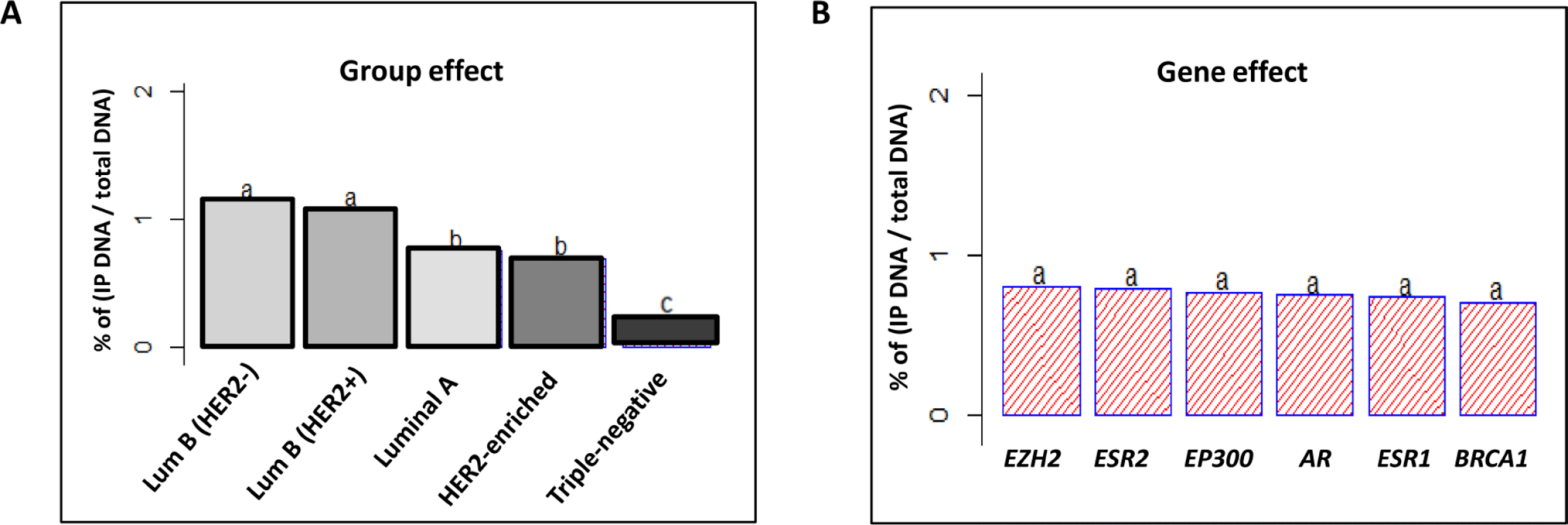
Tukey’s post-hoc comparison of the means analyzing Group and Gene effects ANOVA test followed by Tukey’s multiple comparison test were performed on the results of 50 ChIP assays analyzed by q-PCR (**A**) The statistical analysis discerned 3 different SIRT1 enrichment patterns depending on tumor molecular subtype (Group effect). (**B**) Insignificant discrimination of SIRT1 enrichment in relation to variable gene types (Gene effect). The letters ‘a’, ‘b’ and ‘c’ indicate statistical significance between groups.

In order to determine whether HDAC SIRT1 interacts with histone H3 acetylated epi-marks in human breast cancer, we began by performing chromatin immunoprecipitation (ChIP) assays of SIRT1 on 50 breast tumors and their 50 matched normal tissues (*n* = 10 tumors for each of the 5 molecular subtypes). The assays were then analyzed with real-time quantitative PCR (q-PCR) targeting the promoters of a gene panel consisted of 6 genes. The targeted genes are strongly deregulated and directly implicated in the pathogenesis of breast cancer, as follows: *ERS1, ERS2* and *AR* genes that code for the Estrogen receptors (ER-α), (ER-β) and the Androgen receptor (AR) respectively, the tumor suppressor gene *BRCA1,* and *EZH2* and *EP300* genes coding for histone modifying enzymes (EZH2) and (p300) respectively. The results of ChIP assays showed a significant increase of SIRT1 enrichment on promoters of targeted genes in HRBC and H2BC subtypes, and less significantly in TNBC subtype in comparison to matched normal tissues. The data evoke the possibility that SIRT1 plays a role in the epigenetic regulation of these genes expression in breast cancer (Figure 2). A multi-way analysis of variance (ANOVA) test showed a significant difference of SIRT1 enrichment patterns on gene promoters across all subtypes. Tukey’s range test was then used for multiple comparisons to identify sample means that are significantly different from each other. Two factors were taken into account when performing the statistical procedures: breast cancer molecular subtype (Group effect) and targeted gene type (Gene effect). The post-hoc analysis distinguished 3 distinct patterns of SIRT1 enrichment depending on human breast tumors subtypes, SIRT1 was found to be most enriched on target gene promoters in luminal B subtypes, then luminal A and HER2-enriched subtypes and finally, least enriched in TNBC subtype (Figure 3A). However, there was no significant discrimination of SIRT1 enrichment in relation to different types of genes (Figure 3B).

**Figure 4:**
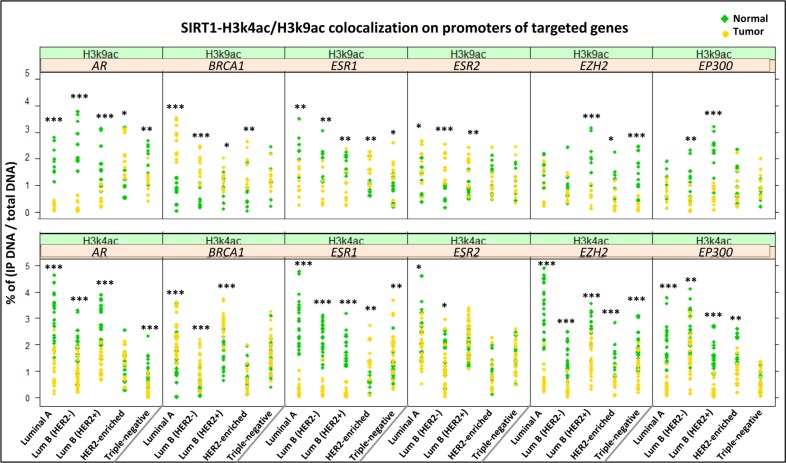
Simultaneous colocalization of SIRT1-H3k4ac and SIRT1-H3k9ac on targeted gene panel promoters across all 5 molecular subtypes versus matched normal tissues Column scatter plot showing the results of ChIP followed by re-ChIP assays using anti-SIRT1, anti-H3k4ac and anti-H3k9ac Abs on 110 breast tumors and their 110 matched normal tissues. The breast tumors were divided as follows: *n* = 26 luminal A, *n* = 24 luminal B (HER2-), *n* = 20 luminal B (HER2+), *n* = 15 HER2-enriched and *n* = 25 triple-negative breast tumors. The efficiency of ChIP was calculated by real time q-PCR on promoters of 6 targeted genes: *AR, BRCA1, ERS1, ERS2, EZH2* and *EP300.* The y-axis represents the percentage of (IP DNA/Total DNA) on target genes promoters. Statistically significant difference of SIRT1 colocalization patterns in tumors versus normal tissues was analyzed by Student’s *t*-test. *P* values were two-tailed, ^*^P < 0.05, ^**^P <0.01 and ^***^P < 0.001 were considered statistically significant.

**Figure 5:**
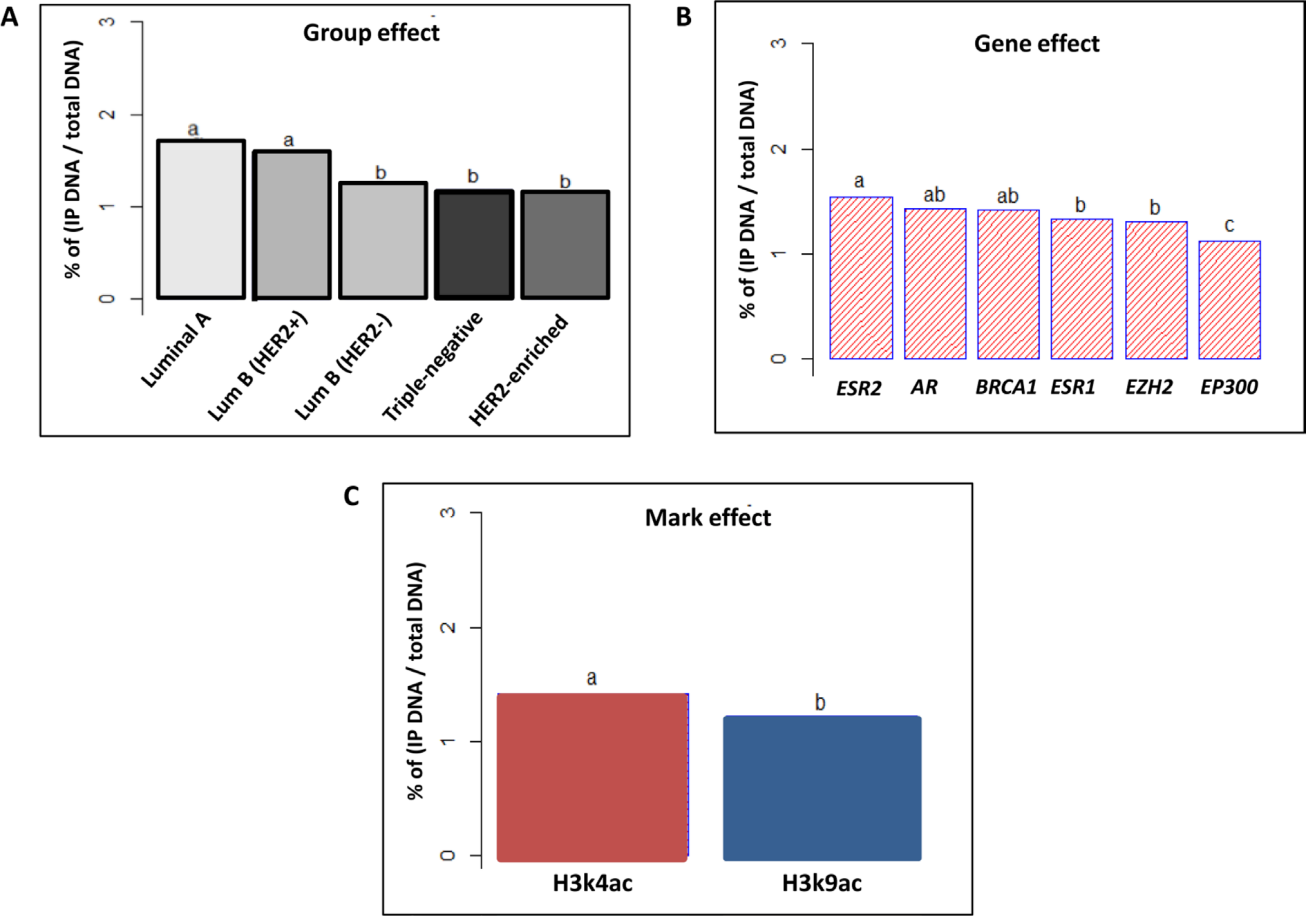
Tukey’s post-hoc comparison of the means analyzing Group, Gene and Mark effects ANOVA test followed by Tukey’s multiple comparison test were performed on the results of 110 ChIP assays analyzed by q-PCR (**A**) The statistical analysis distinguished 2 distinct patterns of SIRT1-H3k4ac/H3k9ac collocation on target promoters depending on tumor molecular subtype (Group effect). (**B**) 3 distinct patterns of SIRT1-H3k4ac/ H3k9ac collocation depending on gene type (Gene effect). (**C**) Significant collocation of SIRT1 with H3k4ac over H3k9ac across all gene types and tumor subtypes (Mark effect). The letters ‘a’, ‘b’ and ‘c’ indicate statistical significance between groups.

**Figure 6:**
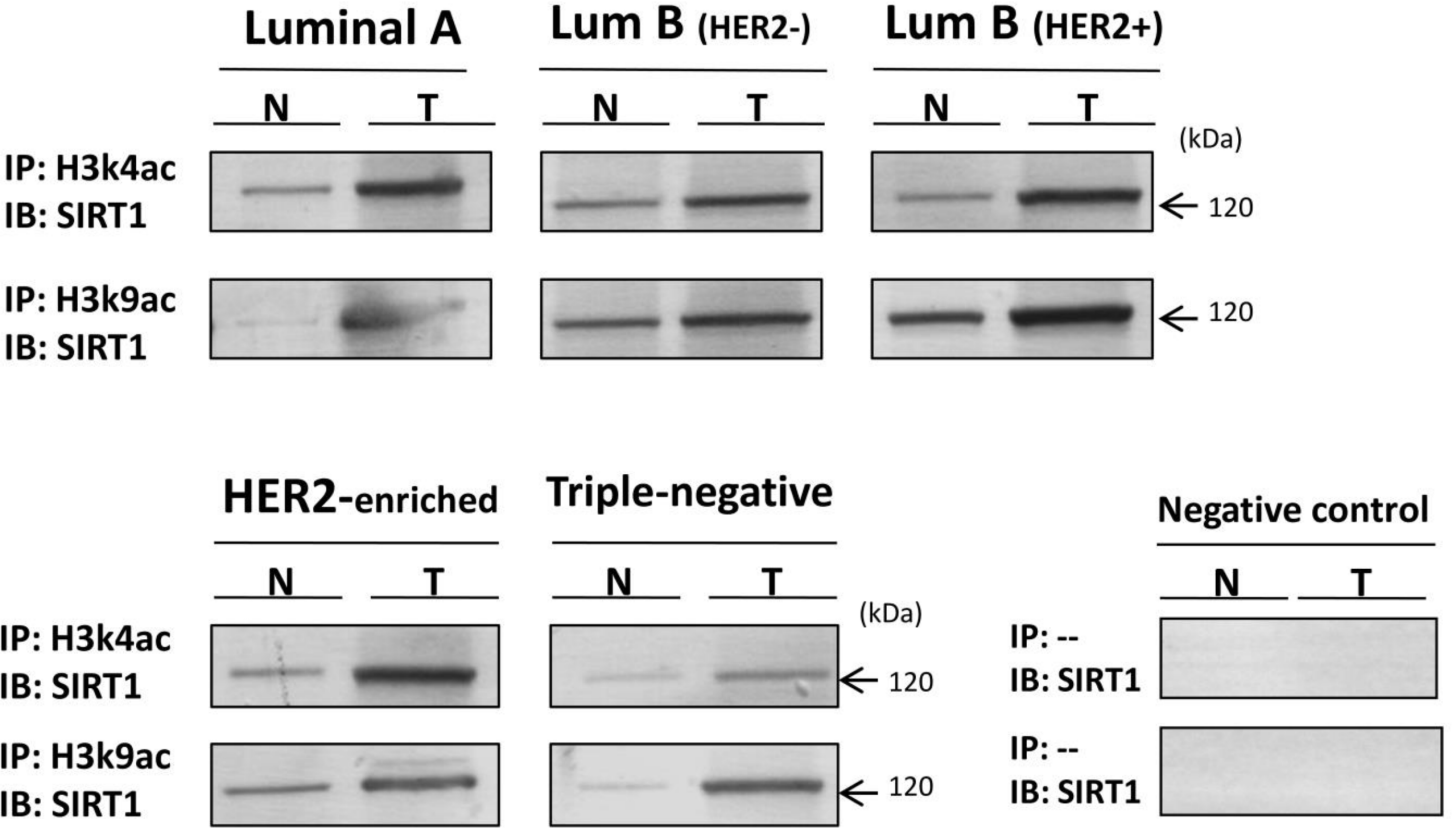
Global physical interaction between SIRT1 and H3k4ac/H3k9ac epi-marks across all molecular breast tumor subtypes compared to matched normal tissues 100 to 500 μg of extracted proteins were at first immunoprecipitated using anti-H3k4ac or anti-H3k9ac Abs (IP), or immunoprecipitated without Ab (IP:--) that served as negative control. The immunoprecipitates were then immunoblotted (IB) with anti-SIRT1 Ab. All experiments were performed in triplicate fashion. N: Normal, T: Tumor.

After confirming the presence of SIRT1 on target gene promoters, we proceeded to investigate whether SIRT1 specifically interacts with histone H3 acetylated marks on those promoters by performing ChIP followed by re-ChIP assays on 110 breast tumors from all 5 molecular subtypes and their 110 matched normal tissues (*n* = 26 luminal A, *n* = 24 luminal B (HER2-), *n* = 20 luminal B (HER2+), *n* = 15 HER2-enriched and *n* = 25 triple-negative). Breast tissues were first assayed by ChIP using anti-H3k4ac or anti-H3k9ac Abs. The obtained samples were then re-immunoprecipitated a second time with anti-SIRT1 Ab. Finally, the immunoprecipitates were analyzed by real-time q-PCR targeting the promoters of the breast cancer-related gene panel previously described. The results showed a simultaneous co-occupancy of SIRT1 with H3k4ac and with H3k9ac on all 6 gene promoters across all 5 subtypes in comparison to matched normal tissues (Figure 4), suggesting that SIRT1 could affect the expression of our targeted genes through epigenetic modification of histone H3 lysine 4 (K4) and lysine 9 (K9) on their promoters. The results also showed a great discrepancy of SIRT1-H3k4ac/H3k9ac colocalization profiles that seem to differ depending on molecular subtype, targeted gene type and studied epi-mark, suggesting that SIRT1- epigenetic regulation depends on multiple factors in different molecular subtypes. To further clarify this observation, multiple-group comparisons ANOVA test followed by Tukey’s range test were carried out with three factors taken into account: molecular subtype (Group effect), gene type (Gene effect) and targeted epi-mark (Mark effect). The post-hoc analysis discerned 2 different SIRT1-H3k4ac/H3k9ac colocalization profiles depending on tumor subtype (Figure 5A) and 3 different colocalization profiles depending on gene type (Figure 5B). Intriguingly, the statistical analysis showed that SIRT1 significantly colocalizes with H3k4ac over H3k9ac on targeted genes across all tumor subtypes (Figure 5C). Afterward, we proceeded to examine whether there is an actual direct interaction between HDAC SIRT1 and histone H3 epi-marks in breast cancer. To do so, we conducted several co-immunoprecipitation assays. Proteins were extracted from breast tumors from each of the 5 molecular subtypes and their matched normal tissues. Extracted proteins were at first immunoprecipitated with anti-H3k4ac or anti-H3k9ac Abs, the immunoprecipitates were then immunoblotted with anti-SIRT1 Ab. The co-immunoprecipitation assays highlighted a global physical interaction between SIRT1 and H3k4ac as well as H3k9ac across all molecular subtypes, implying that SIRT1 could directly deacetylate H3k4ac and H3k9ac in breast cancer. Additionally, the direct interaction between SIRT1 and both epi-marks is significantly increased in breast tumors compared to matched normal tissues (Figure 6).

### Active role of SIRT1 in the deacetylation of H3k4 acetylated mark (H3k4ac) in human breast cancer

Unlike H3k9ac and H4k16ac, H3k4ac is not a known histone target of human histone deacetylase SIRT1. However, Silent Information Regulator 2 (SIR2), the highly conserved orthologue of mammalian SIRT1 in yeast, is the major HDAC of H3k4ac [36]. After uncovering an inverse correlation between SIRT1 and H3k4ac expression patterns, a simultaneous co-occupancy on the same genomic locus and a direct physical interaction between the two across all breast tumors subtypes, we hypothesized that HDAC SIRT1 could play an active role in the deacetylation of H3k4ac in human breast cancer.

### Inverse correlation between SIRT1 and H3k4ac expression patterns in 5 intrinsic subtype breast cancer cell lines

**Figure 7:**
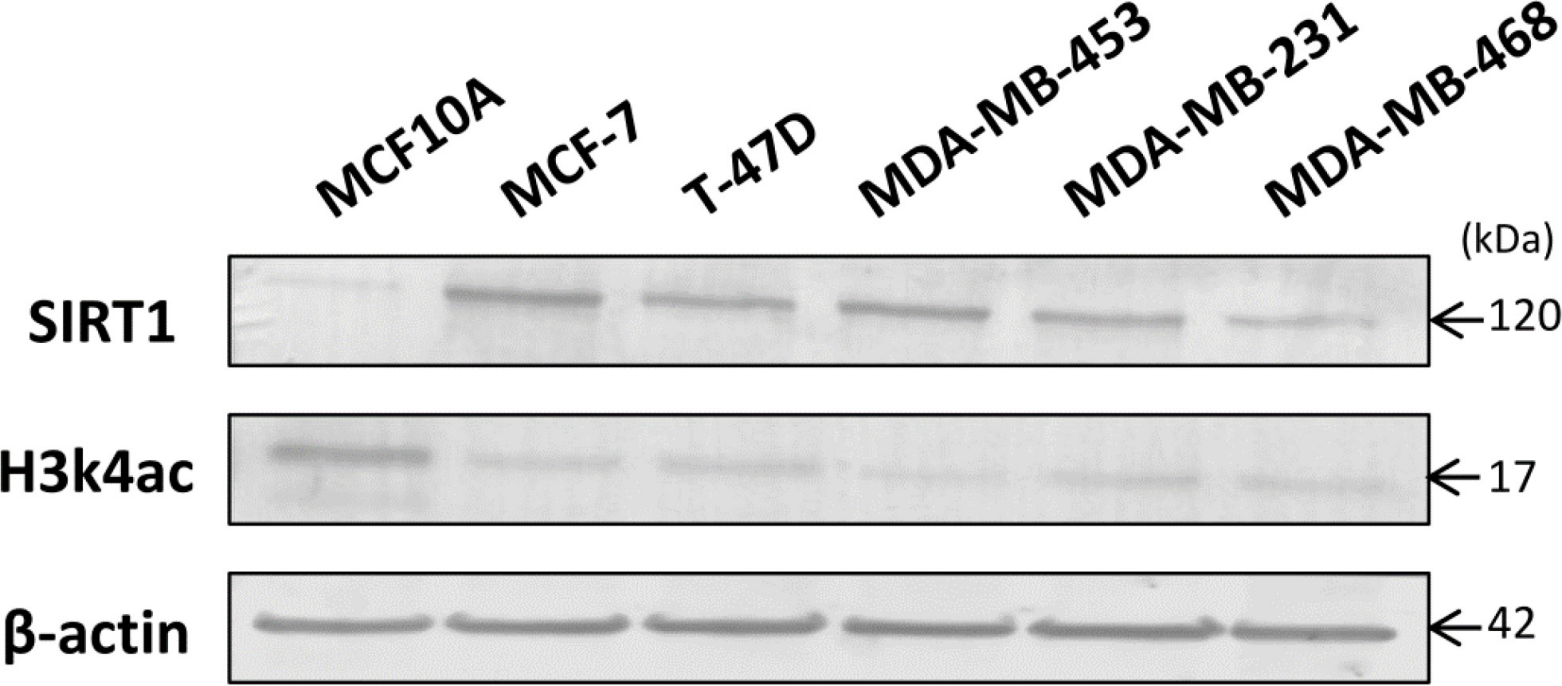
Inverse correlation between SIRT1 and H3k4ac expression levels in 5 intrinsic breast cancer cell lines compared to normal breast cell line Proteins were extracted from MCF10A, MCF-7, T-47D, MDA-MB 453, MDA-MB 231 and MDA-MB 468 cell lines. Equal amounts of extracted proteins were immunoblotted using anti-SIRT1 Ab (120 kDa) and anti-H3k4ac Ab (17 kDa). β-actin (42 kDa) served as an internal loading control.

We began our *in-vitro* experiments by assessing the relative expression levels of SIRT1 and H3k4ac in 5 intrinsic subtype breast cancer cell lines using immunoblot analysis. (ER+) breast cancer cell lines: MCF-7 and T-47D were used as representatives of the luminal subtype, whereas (ER-) breast cancer cell lines: MDA-MB 453, MDA-MB 231 and MDA-MB 468 were used as representatives of the triple-negative subtype. The normal breast cell line MCF10A was used as a control. We observed significantly high expression of SIRT1 in MCF-7, T-47D, MDA-MB 453 and MDA-MB 231 cell lines, and relatively lower expression in MDA-MB 468, all compared to MCF10A cell line. At the opposite, significantly low H3k4ac expression levels were observed in all 5 intrinsic cell lines in comparison with the control cell line (Figure 7).

### SIRT1-siRNA suppresses SIRT1 expression and induces a global increase in H3k4ac, as well as H3k9ac and H4k16ac expression levels in breast cancer cell lines

**Figure 8:**
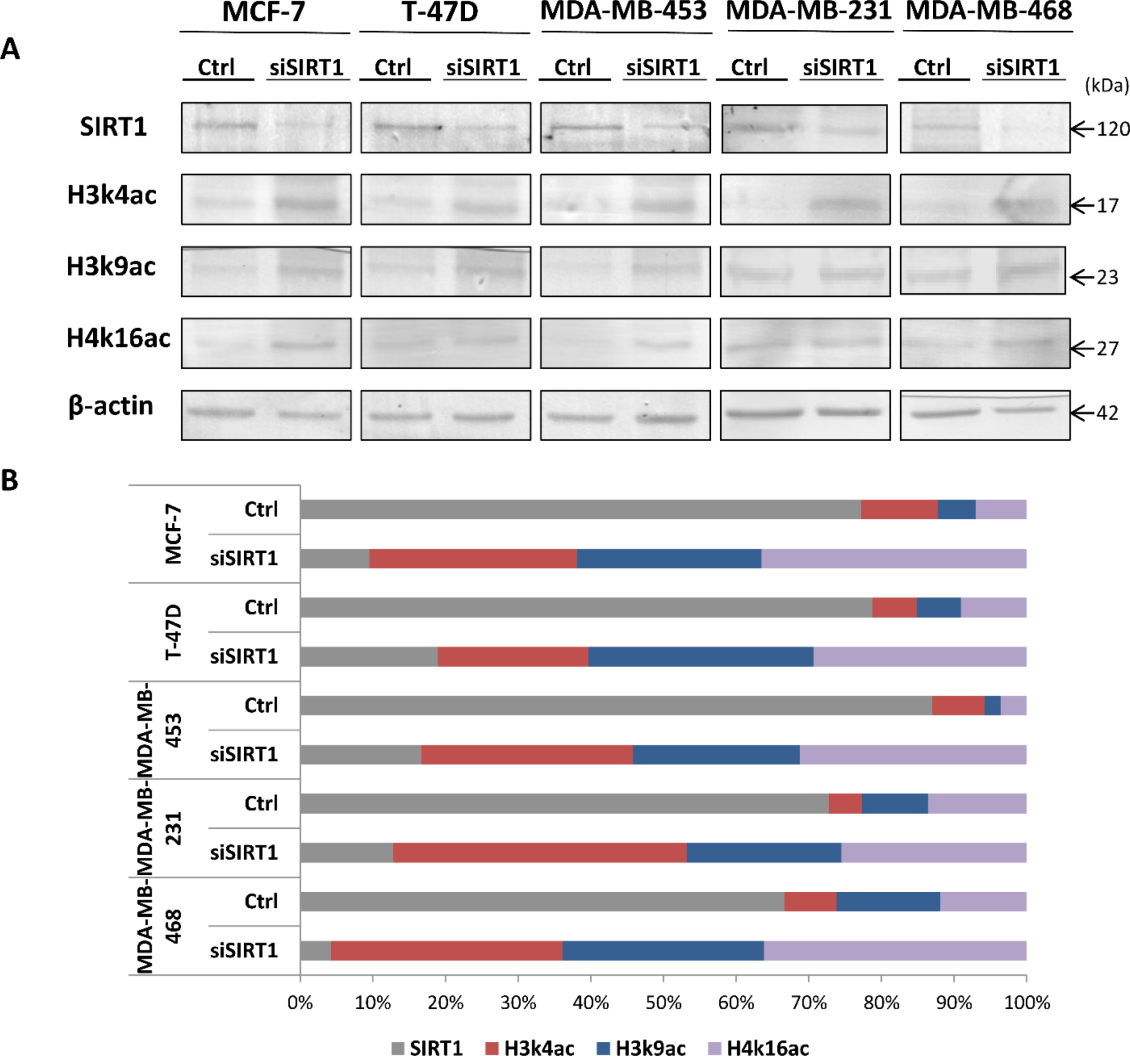
Control of SIRT1 gene silencing with SIRT1-siRNA and its impact on the expression patterns of targeted epi-marks H3k4ac, H3k9ac and H4k16ac *in-vitro* (**A**) MCF-7, T-47D, MDA-MB 453, MDA-MB 231 and MDA-MB 468 cells were transfected with SIRT1-siRNA (siSIRT1) or negative control siRNA (Ctrl). After 48 hours of transfection, equal amounts of proteins were immunoblotted with anti-SIRT1 Ab (120 kDa), anti-H3k4ac Ab (17 kDa), anti-H3k9ac Ab (23 kDa) and anti-H4k16ac Ab (27 kDa). β-actin (42 kDa) served as an internal loading control. (**B**) Relative expression levels were evaluated using Quantity One software and normalized against the internal control β-actin. Each bar represents the percentage contribution of each of the 4 proteins compared to the total set as (100%). All experiments were performed in triplicate fashion.

To gain insight into the mechanism responsible for the deacetylation of H3k4 acetylated mark (H3k4ac), we silenced SIRT1 expression with SIRT1-siRNA (small interfering RNA) in the 5 human-derived mammary cell lines previously described. We were interested in determining whether SIRT1 depletion could alter the relative expression patterns of H3k4ac, as well as H3k9ac and H4k16ac in breast cancer. After 48 hours of transfection, extracted proteins were subjected to immunoblot analysis (Figure 8A). The results showed a significant decrease of SIRT1 expression levels in all MCF-7, T-47D, MDA-MB 453, MDA-MB 231 and MDA-MB 468 transfected cell lines compared to non-transfected control cell lines (Figure 8B). More importantly, a significant increase of H3k4ac, as well as H3k9ac and H4k16ac expression levels, were observed in all 5 transfected cell lines compared to control cell lines (Figure 8B). SIRT1 depletion has led to increased H3k4 acetylation in 5 intrinsic subtype breast cancer cell lines, thus, the deacetylation of H3k4ac seems to be mainly dependent on SIRT1 histone deacetylase activity in breast cancer. Furthermore, the inverse correlation between SIRT1 and the 3 epi-marks expression patterns in transfected versus non-transfected cell lines is similar to that found in breast tumors compared to matched normal tissues. Therefore, SIRT1 seems to be directly responsible for the modulation of H3k4ac, as well as H3k9ac and H4k16ac expression patterns, obviously through direct deacetylation, in breast cancer.

### SIRT1 knockdown modulates histone acetylation at targeted gene panel promoters in a subtype-specific manner

**Figure 9:**
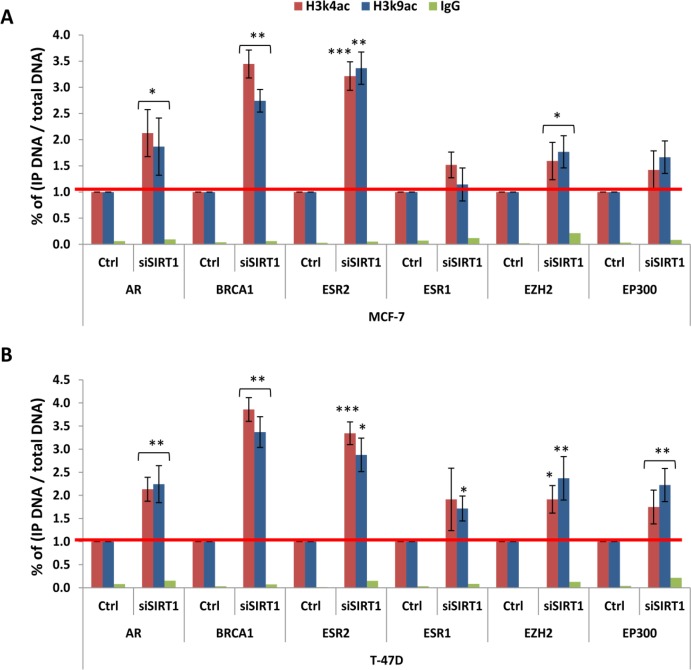
Impact of SIRT1 knockdown on the enrichment of H3k4ac and H3k9ac at targeted gene panel promoters in luminal subtype cell lines Transfected (siSIRT1) and non-transfected (Ctrl) MCF-7 (**A**) and T-47D (**B**) cell lines were subjected to direct ChIP assays using anti-H3k4ac Ab, anti-H3k9ac Ab and non-immune IgG Ab serving as negative control. The efficiency of ChIP was calculated by real time q-PCR on promoters of 6 targeted genes: *AR*, *BRCA1*, *ERS2*, *ERS1*, *EZH2* and *EP300*. All data are presented as fold enrichment of transfected over control cell lines (set as 1). The y-axis represents the percentage of (IP DNA/Total DNA) on target genes promoters. Each column represents the mean ± SD of 3 replicate experiments. *P* values were two-tailed, ^*^P < 0.05, ^**^P < 0.01 and ^***^P < 0.001 were considered statistically significant.

**Figure 10:**
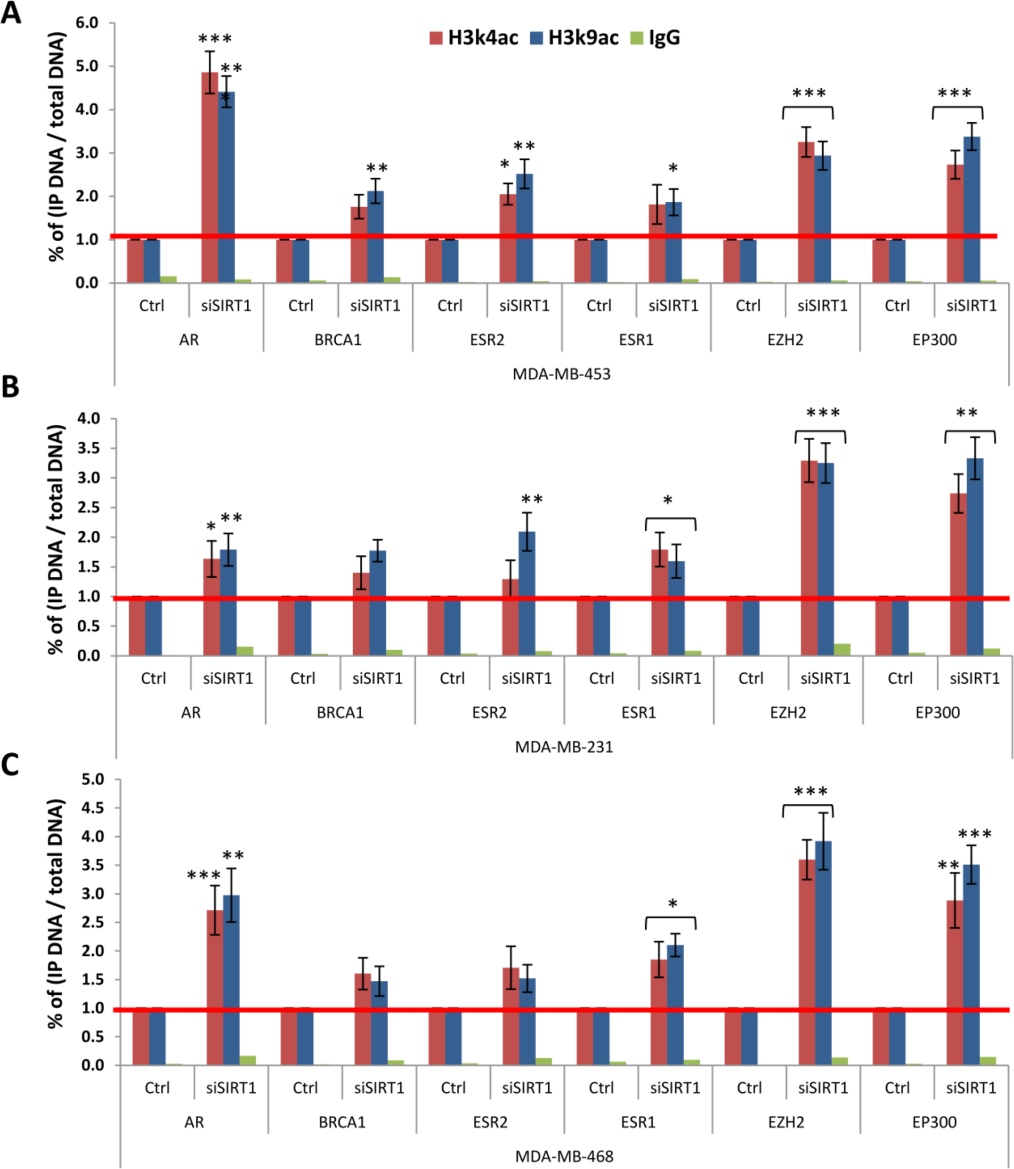
Impact of SIRT1 knockdown on the enrichment of H3k4ac and H3k9ac at targeted gene panel promoters in TNBC subtype cell lines Transfected (siSIRT1) and non-transfected (Ctrl) MDA-MB 453 (**A**), MDA-MB 231 (**B**) and MDA-MB 468 (**C**) cell lines were subjected to direct ChIP assays using anti-H3k4ac Ab, anti-H3k9ac Ab and non-immune IgG Ab serving as negative control. The efficiency of ChIP was calculated by real time q-PCR on promoters of 6 targeted genes: *AR*, *BRCA1*, *ERS2*, *ERS1*, *EZH2* and *EP300*. All data are presented as fold enrichment of transfected over control cell lines (set as 1). The y-axis represents the percentage of (IP DNA/Total DNA) on target genes promoters. Each column represents the mean ± SD of 3 replicate experiments. *P* values were two-tailed, ^*^P < 0.05, ^**^P < 0.01 and ^***^P < 0.001 were considered statistically significant.

To further elucidate SIRT1 epigenetic role in human breast cancer, we conducted direct H3k4ac and H3k9ac ChIP assays on transfected cell lines. We wanted to investigate whether SIRT1 gene silencing could specifically alter the enrichment patterns of histone H3 acetylated epi-marks on the promoters of our target genes, and consequently, impact the targeted genes expression patterns in breast cancer. To explore this possibility, transfected and non-transfected MCF-7, T-47D, MDA-MB 453, MDA-MB 231 and MDA-MB 468 cell lines were subjected to ChIP assays using anti-H3k4ac or anti-H3k9ac Abs. The samples were analyzed by real-time q-PCR targeting the promoters of the breast cancer-related gene panel: *AR*, *BRCA1*, *ERS2*, *ERS1*, *EZH2,* and *EP300*. The results showed a significant increase of both targeted epi-marks H3k4ac and H3k9ac on all 6 gene promoters across all 5 transfected cell lines in comparison to non-transfected control lines (Figures 9 and 10). Interestingly, 2 distinct patterns of H3k4ac and H3k9ac enrichment can be observed following SIRT1 knockdown, the patterns seem to be predominantly dependent on breast cancer cell line intrinsic subtype. The 2 epi-marks were found to be particularly enriched on *BRCA1* and *ESR2* gene promoters in luminal (ER+) subtype cell lines: MCF-7 (Figure 9A) and T-47D (Figure 9B). In contrast, both epi-marks were especially enriched on *AR*, *EZH2* and *EP300* promoters in triple-negative (ER-) subtype cell lines: MDA-MB 453 (Figure 10A), MDA-MB 231 (Figure 10B) and MDA-MB 468 (Figure 10C), implying that SIRT1 regulates its H3 histone targets principally depending on molecular subtype. In conclusion, SIRT1 siRNA-mediated knockdown has significantly increased the acetylation levels of H3k4ac and H3k9ac at the breast cancer-related gene panel promoters; thus, SIRT1 mediates the deacetylation of histone marks H3k4ac, as well as H3k9ac, in breast cancer. The results also revealed SIRT1 differential regulation of H3 acetylated epi-marks in a subtype-specific manner.

## DISCUSSION

Breast cancer is the most commonly diagnosed cancer in women worldwide; it is a multifactorial genetic disease. Sporadic breast tumors represent 85 to 90% of all breast tumors and are especially characterized by an altered epigenome. Deregulated histone epigenome along with other epigenetic alterations play a crucial role in the initiation and progression of breast cancer [9, 37]. Sirtuin-1 is a class III histone deacetylase that can deacetylate both histone and non-histone targets. The mammalian counterpart of yeast SIR2 is deeply implicated in breast cancer development and metastasis. The contradictory functional roles of SIRT1 in breast cancer have been extensively studied over recent years. However, the underlying molecular mechanism by which HDAC SIRT1 regulates its acetylated histone targets, and consequently cancer-related gene expression in breast cancer, is still unknown. In this study, we identified a new prime target of SIRT1 in breast cancer, the acetylated H3k4 histone mark (H3k4ac). We also highlighted a SIRT1-dependent modulation of histones H3 and H4 acetylation patterns in breast cancer. Moreover, we revealed that SIRT1 regulation of its H3 acetylated targets depends greatly on gene type and molecular subtype. Furthermore, we showed that SIRT1 depletion increases histone H3 acetylation levels in a subtype-specific manner at 6 breast cancer-related gene promoters: *AR, BRCA1, ERS1, ERS2, EZH2,* and *EP300,* suggesting that SIRT1 could play an active role in regulating their expression in breast cancer pathogenesis. This is the first report that characterizes the epigenetic behavior of SIRT1 in breast cancer and establishes its status as an epigenetic eraser in human breast carcinoma.

Alteration of histone epigenome is one of the earliest steps in oncogenic transformation. Since histone marks have a direct effect on cancer-related gene expression [7, 38], and since different breast cancer subtypes present distinct gene expression profiles [3] [39], it becomes essential to study the mechanisms of histone epigenome regulation in different subtypes of breast cancer pathogenesis. SIRT1 plays a major role in maintaining genome integrity, largely through regulation of epigenetic mechanisms. SIRT1 epigenetic regulation is realized through direct deacetylation of specific histone markers and controlling the activity of chromatin-modifying enzymes [22]. Histone marks H3k4ac, H3k9ac, and H4k16ac are well-established epigenetic markers of active transcription and actively participate in gene expression [12, 13]. In this study, we showed that the 3 epi-marks relative expression patterns were significantly reduced in breast tumors compared to normal tissues, especially in HRBC and H2BC subtypes. Interestingly, SIRT1 is significantly upregulated in those particular subtypes, previously described in our earlier study [35]. This observation prompted us to suggest that SIRT1 is directly or indirectly responsible for the modulation of the 3 targeted marks in breast cancer. To validate this observation, we silenced SIRT1 expression *in-vitro* via small interfering RNA (siRNA). We opted to use 5 human mammary cell lines that represent the 2 main molecular subtypes of breast cancers: luminal (ER+) and triple-negative (ER-) subtypes. We chose MCF-7 and T-47D cell lines that are classically used as representatives of luminal subtype. Whereas (ER-) cell lines MDA-MB 453, MDA-MB 231 and MDA-MB 468 were used as representatives of the 3 main molecular subtypes of triple-negative breast cancers: Luminal Androgen Receptor (LAR), Mesenchymal-like and Basal-like subtypes respectively, as elegantly characterized by Lehman *et al.* [40]. SIRT1 gene silencing has caused a significant increase of global expression levels of the 3 targeted epi-marks in all transfected lines versus non-transfected control lines, we thus concluded that SIRT1 is actively responsible for the modulation of H3k4ac, H3k9ac, and H4k16ac in luminal and triple-negative molecular subtypes of breast cancer.

To further analyze SIRT1 epigenetic role in sporadic breast cancer, we opted to study SIRT1 interaction with its H3 acetylated targets by carrying out ChIP and re-ChIP assays on 6 breast-cancer related genes: *AR, BRCA1, ERS1, ERS2, EZH2* and *EP300.* The targeted genes play major roles in breast cancer carcinogenesis, either by stimulating breast tumors development and tumor progression, such is the case for oncogenes *AR* [41]*, ERS1* [42]*, EZH2* [43] and *EP300* [44], or having anti-proliferative properties such as tumor suppressor genes *BRCA1* [45] and *ERS2* [46]. SIRT1 ChIP data implies that the latter could have an active role in regulating the expression of these genes in breast cancer either by directly modulating the epigenetic histone markers on their promoters and/or recruiting other chromatin-modifying complexes to that genomic area. However, SIRT1 less significant binding in TNBC subtype could be explained by its reduced expression in it. ChIP, re-ChIP as well as co-immunoprecipitation assays confirmed that SIRT1 physically interacts with and regulates its H3 histone targets H3k4ac and H3k9ac across the 5 molecular subtypes. However, SIRT1 epigenetic regulation is significantly discriminated by gene type and molecular subtype. These results are in line with the findings of Li *et al.* [11] who demonstrated widespread subtype-specific histone modifications in different molecular subtypes. In fact, SIRT1 also negatively regulates the activity of epigenetic ‘writers’ that deposit the histone markers [22]. It has been shown that SIRT1 interacts with and impairs the activity of histone acetyltransferases (HATs) p300 and MOF that are responsible for H3k9 and H4k16 acetylation, [47, 48] respectively. Therefore, SIRT differential epigenetic regulation in breast cancer seems to extend to both histone markers and their epigenetic ‘writers’.

SIRT1 widespread regulation of multiple cancer-related enzymes often leads to its multifaceted functions in various cancers. In consequence, the contradictory roles of SIRT1 were demonstrated in various human malignancies. In colorectal cancer (CRC), both confirmed tumor-suppressive and tumor-promoting functions of SIRT1 have been reported [49]. In a previous study, we suggested a bivalent role of SIRT1 in breast cancer based on its differential expression patterns in human breast tumors. We suggested that SIRT1 most probably has an oncogenic role in HRBC subtypes and a tumor-suppressor role in TNBC subtype [35]. To further explore SIRT1 differential epigenetic regulation in breast cancer, we performed *in-vitro* ChIP analysis with H3k4ac and H3k9ac on SIRT1-siRNA transfected cell lines previously described. SIRT1 knockdown has generated 2 distinct profiles of both epi-marks enrichment on targeted gene promoters that corresponds to the 2 main molecular breast cancer subtypes. The results showed an increase of H3k4ac and H3k9ac expression by 3 to 4-fold on *BRCA1* and *ESR2* genes promoters in both (ER+) cell lines, indicating that SIRT1 contributes to their repression through epigenetic chromatin modification; hence exerting oncogenic properties in breast cancer luminal subtypes. These results are consistent with the findings of Elangovan *et al.* [50] and Ma *et al.* [51] who reported that SIRT1 overexpression in luminal breast cancer subtypes is positively correlated with an oncogenic behavior. At the opposite, in (ER-) cell lines MDA-MB 453, MDA-MB 231 and MDA-MB 468, SIRT1 deficiency induces a 2.5 to 4-fold increase of H3k4ac and H3k9ac expression on *EZH2* and *EP300* promoters, indicating that SIRT1 contributes to the 2 oncogenes repression; hence exerting tumor-suppressive properties in breast cancer triple-negative subtypes. However, a slight increase of H3k4ac and H3k9ac expression on *EZH2* promoter was also observed in the in (ER+) cell lines, implying that *EZH2* expression could be regulated in part by SIRT1 in different subtypes of breast cancer. We also noticed a dramatic increase of H3k4ac and H3k9ac expression by 4.5 to 5-fold on *AR* promoter in MDA-MB 453 cell line representative of the LAR subtype. In fact, LAR subtype or apocrine breast carcinoma is characterized by the expression of *AR* oncogene that contributes to breast tumorigenesis [40]. Therefore, SIRT1 seems to exert tumor-suppressive properties in apocrine breast cancer as well, through epigenetic repression of the *AR* oncogene. These findings are in line with the studies of Yi *et al.* [52] and Simic *et al.* [53] who reported that SIRT1 overexpression suppressed cancer metastasis and tumor cell invasion in triple-negative breast cancer. Based on the above knowledge, we suggest that SIRT1 selectively regulates its histone targets, and consequently gene expression and that SIRT1 epigenetic regulation in breast cancer seems to be predominantly governed by gene type and molecular subtype.

In conclusion, we analyzed an aspect of SIRT1 epigenetic control in breast tumors and established SIRT1 status as an epigenetic eraser in breast cancer. After *ex-vivo* studies on paired breast tumor/normal samples across all molecular subtypes, as well as *in-vitro* experiments on human mammary cell lines, we report that SIRT1 mediates the deacetylation of H3k4ac histone marker in breast cancer. SIRT1 also modulates histones H3 and H4 acetylated marks in different subtypes of breast cancer. In addition, SIRT1 physically interacts with and regulates its H3 acetylated targets in a subtype-specific fashion. Moreover, SIRT1 deficiency is associated with substantial induction of acetylated H3k4 and H3k9 epigenetic marks on 6 breast cancer-related gene promoters: *AR, BRCA1, ERS1, ERS2, EZH2*, and *EP300*. We postulate that SIRT1 plays a differential role in breast cancer development depending on molecular subtype, in part through its epigenetic action. This study thus further consolidates the potential use of SIRT1 as a druggable epigenetic target in human breast cancer.

## MATERIALS AND METHODS

### Patients’ selection and collection of tissue samples

This study included a total of 135 patients admitted to the Centre Jean Perrin from June 2010 to December 2016 for cancer treatment, and diagnosed with breast cancer carcinoma. Patients were informed about the study and gave informed consent prior to inclusion. All 135 tumors and their adjacent normal breast tissues came from the Centre Jean Perrin tumor bank, Biological Resource Center (CRB), accredited under No.BB-0033-00075, where they were stored in liquid nitrogen at −196° C. Patients who received chemotherapy, hormonal therapy and/or radiotherapy for cancer in other parts of the body were excluded from the study, as were patients with predisposition to breast cancer and/or family members with breast cancer.

### Molecular breast cancer subtype classification

Based on estrogen receptor (ER), progesterone receptor (PR), human epidermal growth factor receptor 2 (HER2) and Ki-67 proliferative index; breast tumors were classified into 5 intrinsic subtypes according to the St. Gallen Consensus Conference guidelines [4] as follows: Hormone Receptor-positive Breast Cancer (HRBC) comprising luminal A [ER+, PR+, HER2- and Ki-67 <14%], luminal B (HER2−) [ER+, PR+/−, HER2− and Ki-67 ≥14%] and luminal B (HER2+) [ER+, PR-, HER2+ and any Ki-67]. HER2 Breast Cancer (H2BC): [ER−, PR− and HER2 overexpressed], and lastly Triple-Negative Breast Cancer (TNBC): [ER−, PR−, and HER2−].

### Breast cancer cell lines and cell culture

All 6 human cell lines used in this study were purchased from the ATCC (American Type Culture Collection, Manassas, VA, USA). (ER+) breast cancer cell lines: MCF-7 and T-47D were used as representatives of the luminal subtype. (ER-) breast cancer cell lines: MDA-MB 453, MDA-MB 231 and MDA-MB 468 were used as representatives of the triple-negative subtype. MCF10A, a normal breast cell line, was included as a control. MCF-7 and T-47D cells were cultured in RPMI-1640 medium (Gibco Grand Island, NY) supplemented with 10% fetal bovine serum (Invitrogen, Carlsbad, CA, USA), 1% L-glutamine (Invitrogen, Carlsbad, CA, USA), 0.1% gentamycin (Panpharma, Luitré, France) and insulin (1–4 mg/ml, Novo Nordisk, Denmark) in a humidified atmosphere at 37° C containing 5% CO2. MDA-MB 453, MDA-MB 231 and MDA-MB 468 cells were cultured in Leibovitz’s L-15 medium (Gibco) containing 10% fetal bovine serum, 1% L-glutamine and 0.1% gentamycin in a 37° C humidified atmosphere without CO2. MCF10A cells were grown in DMEM/F-12 meduim (Gibco) containing 10% fetal bovine serum, 1% L-glutamine, 0.1% gentamycin and completed with insulin (10 μg/ml), cholera toxin (100 ng/ml), epidermal growth factor (20 ng/ml), hydrocortisone (500 ng/ml) (Sigma) in a humidified atmosphere at 37° C containing 5% CO2.

### SIRT1-siRNA transfection

Breast cancer cell lines were transfected with human SIRT1 Silencer^®^ Pre-designed and Validated siRNAs and Silencer^®^ Negative Control #1 siRNA (Ambion, Life technologies). The sense and antisense RNA sequences are as follows: 5′-GCUGUACGAGGAGAUAUUUtt-3′ and 5′-AAAUAUCUCCUCGUACAGCtt-3′, respectively. According to the manufacturer’s instructions, cells were transfected at 80% confluence level with 30–60 nM of SIRT1-siRNA or negative control siRNA using the Lipofectamine^®^ RNAiMAX transfection reagent (Invitrogen, Life technologies), that was diluted in Opti-MEM™ Medium (Gibco). SIRT1 knock-down was verified 48 hours after transfection by immunoblotting.

### Chromatin immunoprecipitation (ChIP) and re-ChIP assays

ChIP assays were performed on chromatin extracted from tumors and their matched normal tissues, as well as from transfected cell lines using the Auto iDeal ChIP-seq kit for Histones (C01010171, Diagenode, Seraing, Belgium) according to manufacturer’s instructions. The extracted chromatin was later sonicated for 30 min (30 cycles, 30 s ON/ 30 s OFF) at 4° C with Bioruptor™ sonicator (Diagenode). 3 µg of the following antibodies (Abs) were used: anti-H3k4ac Ab (C15410322), anti-H3k9ac Ab (C15410004) (Diagenode) and non-immune rabbit IgG (Kch-504-250, Diagenode) serving as a negative control, The ChIP was carried out by SX-8X^®^ IP-Star^®^ Compact Automated System (Diagenode). The samples were incubated for 3 h for antibody coating with protein A-coated magnetic beads, then for 10 h at 4° C for immunoprecipitation reaction. Later on, 4 μl of elution buffer iE2 was added to the samples and the input was prepared with 2 μl of extracted chromatin in 100 μl of elution buffer iE1/iE2. The reverse cross-linking was performed for 45 min at 65° C. For Re-ChIP assays, the immunoprecipitated DNA from the first ChIP assay was eluted with elution buffer iE1 containing 10 mM dithiothreitol (DTT) for 30 min at 37° C. The second ChIP assay (re-ChIP) was then carried out using 3 µg of anti-SIRT1 Ab (C15200063, Diagenode). At the end, Immunoprecipitated DNA (IP DNA) and total DNA (input) from both ChIP and Re-ChIP assays were purified by MicroChIP DiaPure Columns (C03040001, Diagenode) and analyzed by real-time qPCR. The quality control and efficacy of all Chip assays performed in this study were verified using positive and negative controls provided in the manufacturer’s kit and according to their instructions (Diagenode). Control of ChIP analysis was performed prior to direct SIRT1 ChIP assays (Supplementary Figure 1) and prior to SIRT1 and H3k4ac/H3k9ac ChIP and re-ChIP assays (Supplementary Figure 2).

### Quantitative real-time PCR method and data analysis

5 µl of IP DNA or total DNA were amplified by real-time qPCR using Taqman Universal PCR Master Mix as per the manufacturer’s protocol using the ABI Prism 7900HT real-time PCR system (AB Applied Biosystems, ThermoFisher Scientific). Real-time PCR was performed in triplicate using 96-well MicroAmp Optical plates (AB Applied Biosystems) with optical adhesive film, at a final reaction volume of 25 μl containing 1X TaqMan Universal PCR Master Mix, 250 nM of probe (AB Applied Biosystems) and 400 nM for each of the forward and reverse primers (Sigma-Aldrich). Primer and probe sequences for *AR, BRCA1, ERS2, ERS1, EZH2,* and *EP300* genes were selected with the help of Primer Express software (ABI), and are as follows:

AR gene, forward primer: 5′-TGCGCCAGCACTTGTTTC-3′; reverse primer: 5′-CACCGCGCGCTAACG-3′; probe: 5′-6FAM-CCAAAGCCACTAGGCAG-MGB-3′; *BRCA1* gene, forward primer: 5′-CCCCGTCCAGGAAGTCTCA-3′; reverse primer: 5′-GCGCGGGAATTACAGATAAATT-3′; probe: 5′-6FAM-CGAGCTCACGCCGCGCAG-TAMRA-3′; *ESR2* gene, forward primer: 5′-GAGAGGCTTTGGGTTTGTCAAAT-3′; reverse primer:5′-CCTCTAGTCCACGGCTTTGC-3′; probe: 5′-6FAM-CAGCAAACGTAACCTCGGGCCCTG-TAMRA-3′; *ESR1* gene, forward primer: 5′-CCCTACATTGGCTTAAACATCA-3′; reverse primer: 5′-TCTTTGGATCGCTCCAAAT-3′; probe: 5′-6FAM-TCCAGGCACAACTC-MGB -3′; *EZH2* gene, forward primer: 5′-CCCTCCAGAAACACAATCAATAGA-3′; reverse primer: 5′-CCGCCTGGTCTGGCTTTAT-3′; probe: 5′-6FAM-CAGAGCAGCTCGACTCT TCCCTCAAACTT-TAMRA-3′; EP300 gene, forward primer: 5′-CGATGGCACAGGTTAGTTTCG-3′; reverse primer: 5′-GCGCACCGAGTAGAAAAGATTAA-3′; probe: 5′-6FAM-CAGCCCCGGCCTTCCACGTT-TAMRA-3′.

The thermal reaction cycles used were 50° C for 2 min, 95° C for 10 min, then 40 cycles of 95° C for 15 sec and 60° C for 1 min. The signal was collected at the endpoint of each cycle (Ct) using an AB Prism 7900 Sequence Detector System (AB Applied Biosystems). ChIP efficiency was calculated and reported as a percentage using the formula: % (IP DNA/Total DNA) = 2^ [(Ct(X% total DNA) – log(X %) /log2) – Ct (IP DNA)] × 100%. 2 is the amplification efficiency, Ct (input) and Ct (ChIP) are threshold values obtained from exponential phase of qPCR for the immunoprecipited DNA sample and input sample respectively, and log(X %) /log2 accounted for the dilution 1/X of the input (Diagenode).

### Protein extraction and immunoblot analysis

Whole protein extracts from frozen tissues and cultured cells were obtained using T-PER™ Tissue Protein Extraction Reagent and RIPA buffer (ThermoFisher Scientific) respectively, containing protease and phosphatase inhibitors cocktail (Sigma Aldrich). 25–40 µg of extracted proteins were resolved by electrophoresis on 8–15% SDS-PAGE sodium dodecyl sulfate polyacrylamide gel (Bio-Rad, Hercules, USA), then electro-transferred onto polyvinylidene difluoride membranes (Immobilon-P, PVDF, 0.45 µm, Merck Millipore) in transfer buffer (25 mM Tris-HCL (pH 7.6), 192 mM glycine, 10% methanol). The membranes were blocked with 5% non-fat milk in 0.1% TBS-tween and later immunoblotted with the following primary Abs: anti-SIRT1 Ab (1/500, C15200063), anti-H3k4ac Ab (1/750, C15410322), anti-H3k9ac Ab (1/1000, C15410004), anti-H4k16ac Ab (1/500, C15200219), all purchased from Diagenode and anti-β-actin Ab (1/5000, CP01, Merck Millipore). Membranes were then washed and incubated with alkaline phosphatase-conjugated secondary Abs: anti-mouse IgG (1/2000, S3721) and anti-rabbit IgG (1/2000, S3738) (Promega, Madison, USA)). Immunolabeling was detected using Western Blue^®^ Stabilized substrate for Alkaline Phosphatase (Promega) at room temperature.

### Co-immunoprecipitation assays

Total proteins were extracted from tumors and matched normal tissues using digestion buffer containing protease and phosphatase inhibitors cocktail (Nuclear complex Co-IP kit, Active Motif, CA, USA) according to the manufacturer’s instructions. 100 to 500 μg of protein lysates were incubated in 500 μl of IP Incubation Buffer overnight at 4° C with 5 μg of the following primary Abs: anti-H3K4ac Ab (C15410322) and anti-H3k9ac Ab (C15410004) (Diagenode). Ab/Extract mixture was then incubated with Ab-binding agarose beads for 1 h at 4° C (Protein G Agarose Columns, Active Motif). Afterwards, the Ab/bead complexes were washed with 500 μl of IP Wash Buffer solution supplemented with or w/o BSA, before being eluted with 25 μl of Reducing Buffer. The immunoprecipitates were then immunoblotted with anti-SIRT1 Ab (1/500, C15200063, Diagenode) as previously described.

### Statistical analysis

Correlation between the clinical parameters of our study groups were examined by chi-square test (χ^2^ test) using SPSS statistics software (SPSS Inc., Chicago, IL). Relative expression levels of SIRT1 protein assayed by immunoblotting were assessed numerically using Quantity One software (Bio-Rad, CA). Multiple-group comparisons were performed by ANOVA using R software (version 3.0.3). Post-hoc comparison of the means was performed using Tukey’s multiple comparison test when the *F*-test was significant (*p* < 0.05). Groups were compared using two-tailed Student’s *t*-test carried out after Fisher’s exact test. All experiments were done at least in triplicate and the results were expressed as mean ± SD. In all cases, statistical significance was set at the following *P*-values: *P < 0.05, **P < 0.01 and ***P < 0.001.

## SUPPLEMENTARY MATERIALS FIGURES


