# Supplementary material for: SIRT1-dependent epigenetic regulation of H3 and H4 histone acetylation in human breast cancer
Source: Oncotarget. 2018 Jul 17;9(55):30661–78. doi: 10.18632/oncotarget.25771 (PMC6078139; doi:10.18632/oncotarget.25771)
Supplement: Supplementary file 1 [file oncotarget-09-30661-s001.pdf]

SIRT1-dependent epigenetic regulation of H3 and H4 histone acetylation in human breast cancer

SUPPLEMENTARY MATERIALS

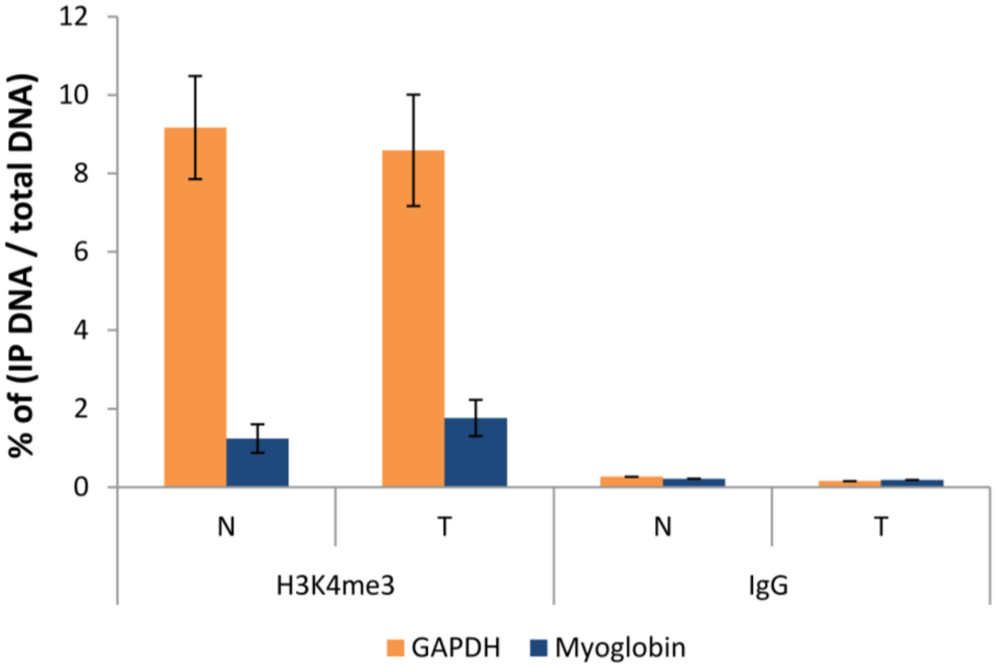

Supplementary Figure 1: Control of ChIP analysis performed on breast tumors and matched normal tissues. ChIP assays were performed on chromatin extracted from breast tumors and matched normal tissues. The assays were carried out using a positive control anti-H34me3 Ab and a negative control non-immune anti-IgG Ab (Diagenode). The efficiency of ChIP was calculated by real time quantitative PCR using the primers of 2 control genes: positive control *GAPDH* and negative control *Myoglobin*. Data are the average of 3 independent experiments and represented as means ± S.D. N: Normal, T: Tumor.

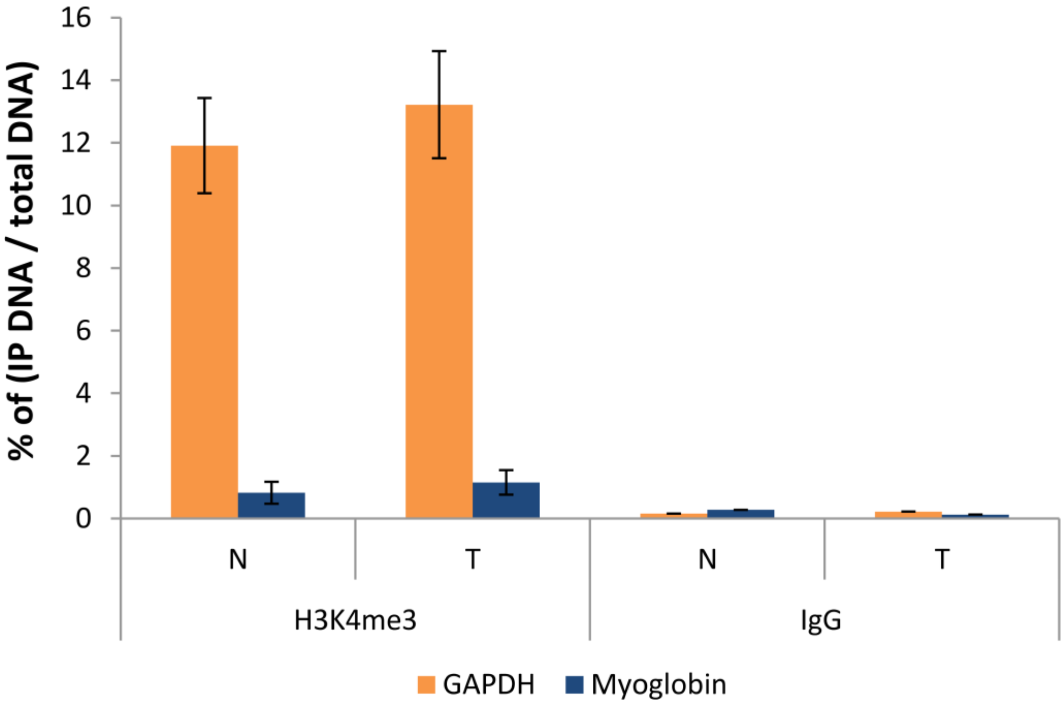

Supplementary Figure 2: Control of ChIP analysis performed on breast tumors and matched normal tissues. ChIP assays were performed on chromatin extracted from breast tumors and matched normal tissues. The assays were carried out using a positive control anti-H34me3 Ab and a negative control non-immune anti-IgG Ab (Diagenode). The efficiency of ChIP was calculated by real time quantitative PCR using the primers of 2 control genes: positive control *GAPDH* and negative control *Myoglobin*. Data are the average of 5 independent experiments and represented as means ± S.D. N: Normal, T: Tumor.
